# Effect of Duty Cycle on Cutting Force for Ultrasonic Vibration-Assisted Milling Carbon Fiber-Reinforced Polymer Laminates

**DOI:** 10.3390/ma16237457

**Published:** 2023-11-30

**Authors:** Yukun Zhang, Junxue Ren, Jinhua Zhou

**Affiliations:** 1 Key Laboratory of High Performance Manufacturing for Aero Engine, Ministry of Industry and Information Technology, School of Mechanical Engineering, Northwestern Polytechnical University, Xi’an 710072, China; ykzhang@mail.nwpu.edu.cn (Y.Z.); rjx1968@nwpu.edu.cn (J.R.); 2 Engineering Research Center of Advanced Manufacturing Technology for Aero Engine, Ministry of Education, School of Mechanical Engineering, Northwestern Polytechnical University, Xi’an 710072, China

**Keywords:** CFRP, ultrasonic vibration-assisted machining, duty cycle, cutting force

## Abstract

Cutting force is an important factor that affects the surface quality of machining carbon fiber-reinforced polymer (CFRP). High cutting force can lead to surface damage such as the burrs and the delamination in the machining process of CFRP. Ultrasonic vibration-assisted machining (UVAM) can reduce the cutting force in the machining process. This work is focused on the relationship between the duty cycle and the cutting force in UVAM of CFRP. Based on the kinematics of UVAM, the movement of the cutting tool edge and the tool–workpiece separation in UVAM were analyzed, and a calculation formula for the duty cycle was obtained. The milling experiment of CFRP was conducted to compare the cutting force between UVAM and conventional machining (CM), and the relationship between the reduction in the cutting force in UVAM and the duty cycle was determined. The experimental results showed that when the duty cycle was 0.2916, the cutting force of UVAM was reduced by 7.4% to 27% compared with that of CM. When the duty cycle was 1, the cutting force of UVAM was reduced by −4.5% to 7.5% compared with that of CM. Therefore, the effect of reducing the cutting force of UVAM can be enhanced by adjusting the process parameters to reduce the duty cycle of UVAM, and a lower cutting force can be obtained.

## 1. Introduction

Ultrasonic vibration-assisted machining (UVAM) is a machining process that combines ultrasonic vibration with conventional machining (CM) by attaching high-frequency vibration to the tool or workpiece. Compared with CM, UVAM with appropriate process parameters has the advantages of reducing cutting forces [1,2,3], lowering cutting temperatures [4], decreasing surface roughness [5,6], and improving tool life [7,8]. Carbon fiber reinforced polymer (CFRP) is a heterogeneous anisotropic material widely used in the aerospace industry due to its high specific strength, high specific modulus, and corrosion resistance. Typically, CFRP requires machining such as milling or grinding after the forming process. However, when cutting CFRP, problems exist such as a short tool life and easy machining damage [9]. Based on the advantages of UVAM, some researchers have conducted research on machining CFRP by using UVAM and compared it with CM. The results demonstrate that using UVAM to process CFRP can reduce cutting force [10,11], lower cutting temperature [12,13], reduce surface roughness [14,15], and improve tool life [16].

During the machining process, ultrasonic vibration changes the motion trajectory of the tool or workpiece, which may cause periodic separation between the tool and workpiece. High spindle speed, low ultrasonic frequency, or low ultrasonic amplitude, may lead to the disappearance of tool–workpiece separation. Brehl et al. [17] used the duty cycle to explain tool–workpiece separation in UVAM. The duty cycle refers to the ratio of the time when the tool contacts the workpiece to the total machining time, and they stated that a decrease in the duty cycle will reduce the cutting force. Chen et al. [18] proposed that different vibration modes result in different separation situations, categorizing the tool–workpiece separation process in one-dimensional and two-dimensional UVAM into three types. Ni et al. [19] presented an analytical model to calculate the tool–workpiece contact ratio (TWCR) for UVAM. They stated that a decrease in the TWCR corresponds to a reduction in net cutting time, which leads to lower cutting force, reduced accumulation of cutting heat, improved surface quality, and extended tool life. Sui et al. [20] found that the tool–workpiece separation and the duty cycle calculation in high-speed ultrasonic vibration cutting (HUVC) are different from the other UVAM methods. The tool–workpiece separation of HUVC depends on the feed rate, the amplitude of ultrasonic vibration, and the fraction part of the cutting frequency ratio, and the duty cycle can be changed by adjusting the spindle speed to alter the phase difference. At present, many references mention the duty cycle of UVAM. However, no existing reference has been found that investigates the relationship between the duty cycle and cutting force in UVAM.

Fiber-reinforced polymers are widely used in various fields such as automotive [21], aerospace [22], and construction [23] due to their excellent material properties. However, this also brings challenges such as difficult processing and surface damage. In the 1980s, some researchers verified the feasibility of using UVAM to process fiber-reinforced composites [24]. They stated that UVAM can reduce the average cutting force and surface roughness and reduce burrs and subsurface damage. Since then, researchers have conducted theoretical and experimental studies on machining CFRP using UVAM. Xu et al. [25,26,27] found that fiber orientation can significantly affect the subsurface damage and cutting force. At the same time, elliptical vibration-assisted cutting can reduce the effect of fiber orientation, and the subsurface damage of elliptical vibration-assisted cutting of carbon fiber composites is less than that of conventional cutting. Yuan et al. [28] developed a cutting force model for rotary ultrasonic drilling of CFRP-T700 based on brittle fracture material mechanism and found that feed rate and spindle speed are the main parameters affecting cutting force. Abd Halim et al. [29] compared UVAM and CM machining CFRP based on experiments. They found that UVAM results in lower maximum cutting forces but faster tool wear, the surface roughness was slightly worse, and resin degradation occurred in both machining methods. At present, there are many studies on using UVAM to reduce the cutting force on CFRP, but the cutting force reduction rate by using UVAM is different in various references. How to adjust process parameters to prevent UVAM from being unable to achieve the effect of reducing cutting force, or to make UVAM achieve a better effect of reducing cutting force, is an issue that needs to be considered when applying UVAM in practical machining.

To address the challenges above, this study started from the tool–workpiece separation characteristic of UVAM, investigated the influence of process parameters on duty cycle and cutting force through theoretical calculations and experiments, and explored the relationship between the duty cycle and the cutting force. First, the relative motion trajectory of the tool and workpiece in UVAM milling was analyzed, the calculation method of the duty cycle was obtained, and the influence factors of duty cycle are discussed. Second, a CFRP cutting force experiment was carried out to compare the cutting force of UVAM and CM, and the impact of process parameters on the UVAM cutting force reduction effect was analyzed. Finally, based on the theoretical study of the duty cycle and the experimental results of cutting force, the influence of the duty cycle on the UVAM cutting force was obtained. This study provides theoretical support for the optimization of UVAM process parameters and determines the process parameter conditions suitable for UVAM, which ensures that UVAM can achieve an effective reduction in cutting force.

## 2. Theoretical Calculation of Duty Cycle for UVAM

### 2.1. Kinematic Analysis of Tool Motion Trajectory

**Figure 1:**
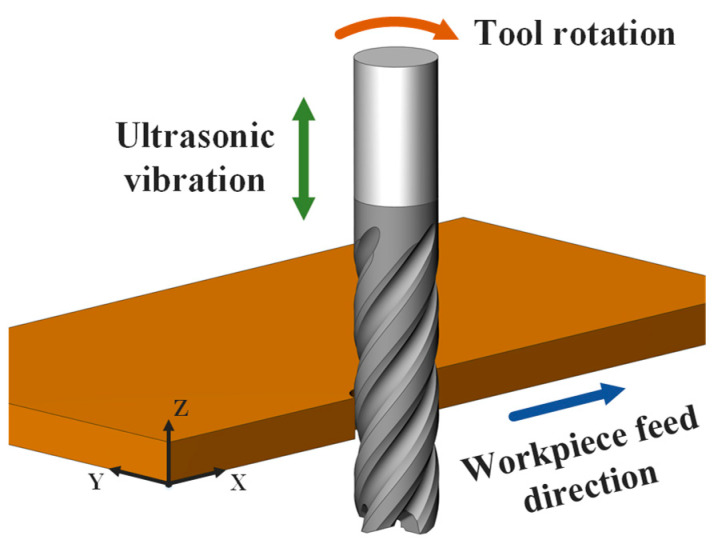
Movement of tool and workpiece in UVAM.

The motion of UVAM milling CFRP laminates is shown in Figure 1. The workpiece feed direction is parallel to the *x*-axis, the tool rotational direction is always in the XY plane, and the ultrasonic vibration direction is parallel to the *z*-axis. In this configuration, the motion trajectory of any point on the tool can be expressed as

(1) $$\left\{\begin{array}{l} x=v_{f}\cdot t+\frac{D}{2}\cdot sin\left[\frac{\pi n}{30}t-\frac{2\pi \left(Z_{i}-1\right)}{Z}\right]\\ y=\frac{D}{2}\cdot cos\left[\frac{\pi n}{30}t-\frac{2\pi \left(Z_{i}-1\right)}{Z}\right]\\ z=a_{p}\cdot sin\left(2\pi f_{z}t\right) \end{array}\right.$$

while D is the tool diameter, n is the spindle speed, t is the time, $v_{f}$ is the feed rate, Z is the number of tool teeth, i is the *i*-th tool teeth, $a_{p}$ is the ultrasonic amplitude, and $f_{z}$ is the ultrasonic frequency.

**Figure 2:**
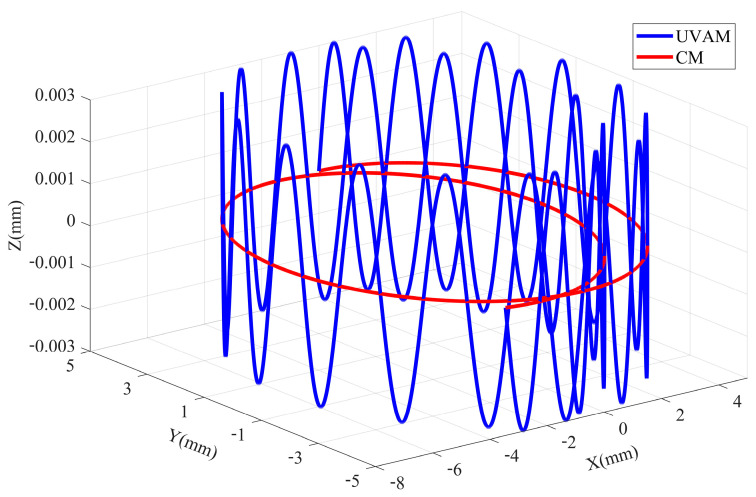
Comparison of the UVAM and the CM tool trajectories.

**Figure 3:**
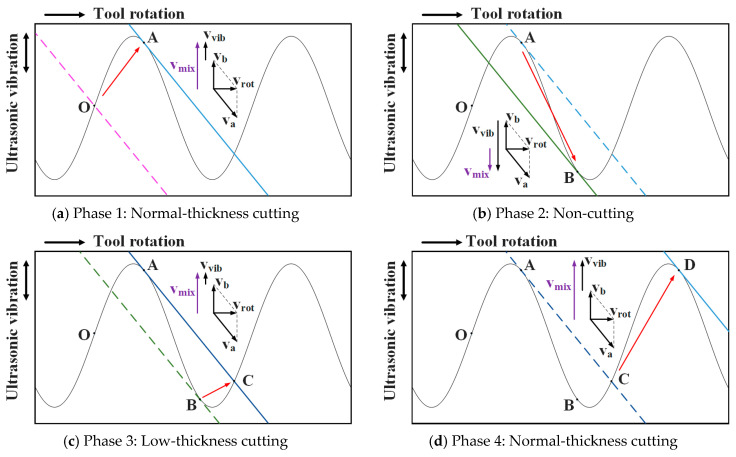
Motion of UVAM in two-dimensional space.

Figure 2 shows the machining trajectory at any point on the tool relative to the workpiece in UVAM and CM. It can be observed that axial ultrasonic vibration significantly changes the motion trajectory of the point, changing the machining trajectory from a planar curve in CM to a more complex three-dimensional curve in UVAM. In CM, the motion trajectories of points on a cutting edge are distributed in different planes, so the motion trajectories of each point will not overlap. However, in UVAM, the motion trajectories of points on the tool edge are all located in the same three-dimensional space, so the trajectories of different points may overlap. To facilitate the kinematic analysis, the cutting space is unfolded into a two-dimensional space, where the helical curve of the cutting edge becomes a straight line, and the rotational motion of the tool becomes linear motion. Figure 3 shows the motion of the tool edge relative to the workpiece in UVAM in two-dimensional space, and the sinusoidal curve in the figure is the motion trajectory of a point on the tool edge. The tool rotation speed $v_{rot}$ is decomposed into $v_{a}$ and $v_{b}$, where $v_{a}$ is parallel to the direction of the tool edge, and $v_{b}$ is parallel to the direction of the ultrasonic vibration velocity. The velocity $v_{mix}$ can be calculated as follows:

(2) $$v_{mix}=v_{vib}+v_{b}$$

From Figure 3, it can be seen that when the tool edge moves along the curve OA, the velocity $v_{mix}$ is in the same direction as $v_{b}$. In this case, the tool performs normal-thickness cutting. When the cutting edge moves along the curve AB, $v_{mix}$ is in the opposite direction to $v_{b}$. In this situation, the tool edge and the workpiece are separated, and no cutting is performed. When the tool edge moves along the curve BC, $v_{mix}$ is in the same direction as $v_{b}$ again. Since OA has already been processed, the tool edge moves along curve BC, resulting in a very small chip thickness. When the tool edge moves along the curve CD, the chip thickness returns to the normal state, and it can be observed that within one ultrasonic vibration cycle, the tool edge goes through three processes, including normal-thickness cutting, non-cutting, and low-thickness cutting. This cycle repeats continuously throughout the entire machining process.

### 2.2. Theoretical Investigation of Duty Cycle

**Figure 4:**
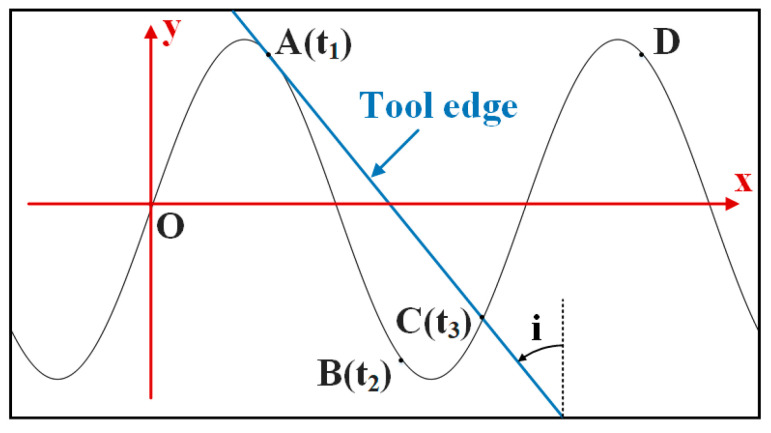
Coordinate system with O as origin.

To study the contact separation situation between the tool and the workpiece in UVAM, it is necessary to obtain the times *t*_1_, *t*_2_, and *t*_3_ for the tool to move to points A, B, and C, respectively. Since the feed rate is typically much smaller than the tool edge rotation speed and the speed of ultrasonic vibration, the influence of the feed rate can be neglected in the two-dimensional analysis. As shown in Figure 4, an xOy coordinate system is established, where the angle i is the helix angle of the tool. At this time, the equation for the motion trajectory of a point on the tool edge in the two-dimensional space is given by

(3) $$\left\{\begin{array}{l} x=\frac{\pi Dn}{60}t\\ y=a_{p}\cdot sin\left(2\pi f_{z}t\right) \end{array}\right.$$

The velocity of the tool edge along the x and y directions is

(4) $$\left\{\begin{array}{l} v_{rot}=\frac{\pi Dn}{60}\\ v_{vib}=2\pi f_{z}a_{p}\cdot cos\left(2\pi f_{z}t\right) \end{array}\right.$$

From the previous analysis, it can be seen that the tool–workpiece separation is related to the tool rotation speed, ultrasonic vibration speed, and tool helix angle. Therefore, the critical condition for tool-workpiece separation and contact is given by

(5) $$v_{vib}=-v_{b}=-\frac{v_{rot}}{tani}$$

The term $-v_{b}$ represents that $v_{b}$ is in the opposite direction to $v_{vib}$. When $v_{vib}<-v_{b}$, the tool and the workpiece are in the separation state. When $v_{vib}>-v_{b}$, the tool and workpiece are in contact and the cutting state. Combining Equations (4) and (5), the calculation formulas for the times *t*_1_, *t*_2_, and *t*_3_ for the tool to reach points A, B, and C are

(6) $$t_{1}=\frac{arccos\left(\frac{-Dn}{120f_{z}a_{p}\cdot tani}\right)}{2\pi f_{z}}$$

(7) $$t_{2}=\frac{1}{f_{z}}-\frac{\arccos\left(\frac{-Dn}{120f_{z}a_{p}\cdot tani}\right)}{2\pi f_{z}}$$

(8) $$\begin{array}{cc} a_{p}\sin\left(2\pi f_{z}t_{3}\right) & =a_{p}\sin\left(\arccos\left(\frac{-Dn}{120f_{z}a_{p}\cdot tani}\right)\right)-\frac{\pi Dn}{60tani}t_{3}\\ & +\frac{Dn\cdot \arccos\left(\frac{-Dn}{120f_{z}a_{p}\cdot tani}\right)}{120f_{z}tani} \end{array}$$

Equation (8) needs to be solved numerically. After obtaining the values of *t*_1_, *t*_2_, and *t*_3_, the times for normal-thickness cutting, non-cutting, and low-thickness cutting can be calculated, and the duty cycle of UVAM can be calculated. The duty cycle refers to the proportion of the time the tool is in contact with the workpiece to the total machining time. Since the low-thickness cutting phase occurs on the machined surface, and the chip thickness during this phase is much smaller than that during the normal-thickness cutting phase, the period from *t*_1_ to *t*_3_ is defined as the time when the tool does not cut. In this case, the UVAM duty cycle DC is calculated as

(9) $$DC=\frac{T-\left(t_{3}-t_{1}\right)}{T}$$

From the critical condition of tool–workpiece separation and contact, it can be seen that when the maximum speed of ultrasonic vibration $v_{vibmax}>v_{rot}/tani$, tool–workpiece separation occurs in machining, and when $v_{vibmax}<v_{rot}/tani$, there is no tool–workpiece separation. From Equation (9), it can be seen that the duty cycle $DC\in \left[0,1\right]$. When DC=0, the tool and workpiece are always separated, and no cutting occurs. When DC=1, the tool is always in contact with the workpiece, continuously in the cutting state. The duty cycle of CM is always DC=1. Therefore, when $v_{vibmax}>v_{rot}/tani$, 0≤DC<1, and only when $v_{rot}=0$ or i=π/2, *DC* = 0. When $v_{vibmax}<v_{rot}/tani$, DC=1.

### 2.3. Effect of Process Parameters on the Duty Cycle

**Figure 5:**
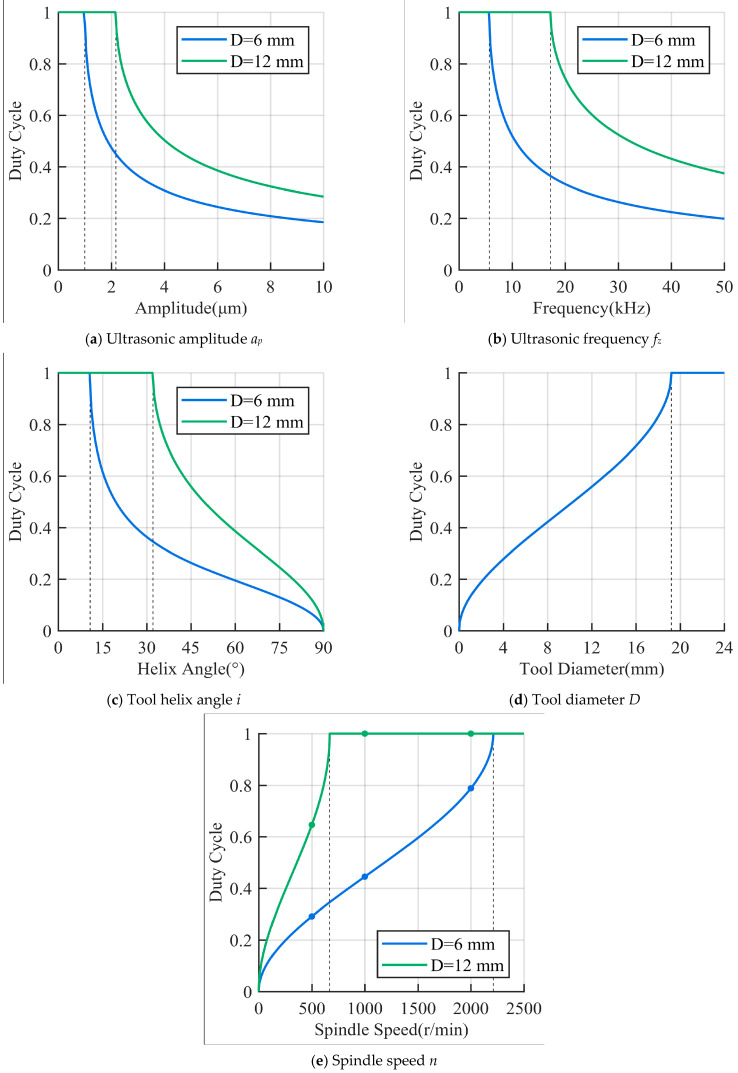
Effect of process parameters on the duty cycle. (**a**–**c**) duty cycle decreases as amplitude, frequency, and helix angle increase, (**d**,**e**) duty cycle increases as tool diameter and spindle speed increase.

According to Equations (6), (8) and (9), the duty cycle DC is related to five parameters: tool helix angle i, ultrasonic amplitude $a_{p}$, ultrasonic frequency $f_{z}$, tool diameter D, and spindle speed n. The relationship between the process parameters and the duty cycle is shown in Figure 5. It can be observed that the duty cycle gradually decreases with the increase in the tool helix angle, ultrasonic amplitude, and ultrasonic frequency, while it increases with the increase in the tool diameter and spindle speed. From Figure 5a,b, it can be seen that when the ultrasonic amplitude and frequency are below the critical value, the duty cycle DC=1. When they exceed the critical value, the duty cycle rapidly decreases with increasing parameters, and then the rate of decrease gradually slows down. In Figure 5c, it can be observed that the duty cycle decreases as the tool helix angle increases; when the helix angle i=π/2, the duty cycle DC=0. Figure 5d,e show that as the tool diameter and spindle speed increase, the duty cycle gradually increases until the duty cycle DC=1. Therefore, to achieve tool–workpiece separation in UVAM, the helix angle, ultrasonic amplitude, and frequency need to exceed critical values, while the tool diameter and spindle speed should be controlled below critical values. Moreover, it can be considered to increase the tool helix angle, ultrasonic amplitude, or frequency and decrease the tool diameter or spindle speed to increase the tool–workpiece separation time, i.e., to reduce the duty cycle.

## 3. UVAM Cutting Force Experiment

In this experiment, the cutting forces of UVAM under different process parameters were measured and compared with the cutting force of CM. Thus, the effect of UVAM in reducing the cutting force in CFRP machining was verified, and the relationship between the duty cycle and the cutting force in UVAM was determined. The workpiece used in this experiment was a multidirectional CFRP laminate with a thickness of 4.3 mm, and the fiber used in the laminate was T800 carbon fiber. The tools were diamond-coated carbide end mills with tool diameters of 6 mm and 12 mm and a tool helix angle of 40°. Both tools were experimented with according to the process parameters given in Table 1, and each set of experiments was conducted to compare the cutting forces of UVAM and CM. In this experiment, the axial depth of cut $a_{p}=4.3\;\mathrm{mm}$, the feed rate range was 50~200 mm/min, the radial depth of cut range was 0.3~1 mm, and the spindle speed range was 500~3000 n/min. The specific experimental design is shown in Table 1.

**Figure 6:**
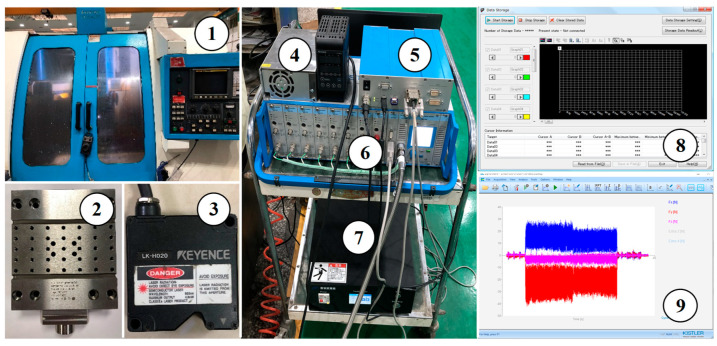
Cutting force experimental equipment and measurement software. (1) VMC850 machine tool, (2) Kistler 9119A multicomponent dynamometer, (3) Keyence LK-H020 sensor head, (4) Keyence LK-G5000 laser displacement sensor, (5) Kistler 5697A data acquisition system, (6) Kistler 5080A charge amplifier, (7) ultrasonic generator, (8) LK-Navigator2 (version 01.04.0.3), and (9) Dynoware software.

The experiment was conducted on the Johnford VMC850 machine tool equipped with an axial ultrasonic vibration tool holder. Before the experiment, the displacement of the tool under ultrasonic vibration was measured using the Keyence LK-G5000 laser displacement sensor with the Keyence LK-H020 sensor head. The displacement sampling frequency was 200 kHz, and the displacement data were processed using the Fourier transform to obtain the ultrasonic frequency and amplitude of UVAM. The cutting force measurement system used during the machining process consisted of the Kistler 9119A multicomponent dynamometer, the Kistler 5080A charge amplifier, and the Kistler 5697A data acquisition system. The cutting force data were displayed and stored through the Dynoware (version 3.3.2.0). The sampling frequency for the cutting force was 6000 Hz, and low-pass filtering was used to process the cutting force data. Figure 6 shows the cutting force experimental equipment and measurement software.

According to the measurements, the ultrasonic frequency of the 6 mm diameter milling tool was found to be 29,932 Hz, with an amplitude of 4.4 μm. The ultrasonic frequency of the 12 mm diameter milling tool was measured to be 27,467 Hz, with an amplitude of 2.9 μm. The cutting force measurement results are shown in Table 2. Compared with CM, the cutting force using UVAM for machining showed a different reduction rate.

## 4. Results and Discussion

**Figure 7:**
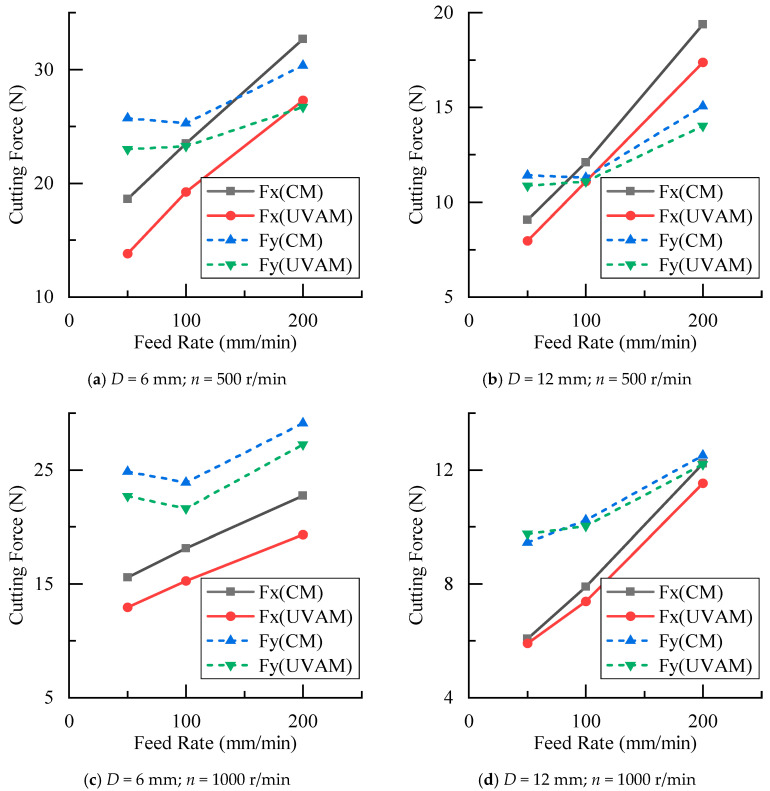
Comparison of cutting forces between CM and UVAM at different feed rates.

The experimental results of the cutting force at different feed rates are shown in Figure 7. As the feed rate increased, both CM and UVAM exhibited a gradual increase in cutting forces. The UVAM cutting force was lower than the CM cutting force under the same process parameters, which proved that the application of ultrasonic vibration reduced the cutting force in milling CFRP. To study the reduction effect of ultrasonic vibration on the cutting force, the reduction rate in the cutting force of UVAM compared with that of CM was calculated as follows:

(10) $$\delta F=\frac{F\left(CM\right)-F\left(UVAM\right)}{F\left(CM\right)}\times 100$$

**Figure 8:**
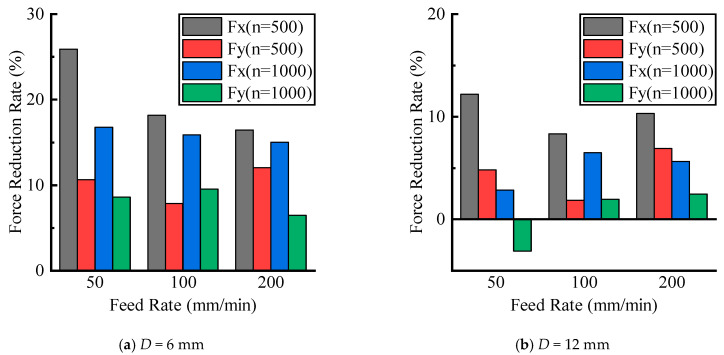
Effect of feed rate on cutting force reduction.

The calculation results are shown in Figure 8, where the *y*-axis represents δF, which indicates the reduction rate of cutting force of UVAM compared with that of CM under the same process parameters. In Figure 8, the average cutting force reduction rate for the D=6 mm tool was as follows: at n=500 r/min, $\delta F_{x}=20.2\%,$ and $\delta F_{y}=10.2\%$; at n=1000 r/min, $\delta F_{x}=15.9\%$, and $\delta F_{y}=8.2\%$. For the D=12 mm tool, the average reduction rate was as follows: at n=500 r/min, $\delta F_{x}=10.3\%$, and $\delta F_{y}=4.5\%$; at n=1000 r/min, $\delta F_{x}=5.0\%$, and $\delta F_{y}=0.4\%$. It can be seen that the cutting force reduction rate for the D=6 mm tool was higher than that of the D=12 mm tool. Additionally, at the same tool diameter, the reduction rate at n=500 r/min was higher than that at n=1000 r/min. According to the calculated duty cycle shown in Figure 5, the duty cycle of the D=6 mm tool was 0.2916 and 0.445, and that of the D=6 mm tool was 0.6467 and 1. This indicated that the value of the duty cycle was inversely proportional to the cutting force reduction rate. Therefore, using a smaller tool diameter and lower spindle speed to achieve a lower duty cycle can enhance the effect of UVAM in reducing the cutting force.

According to Figure 7 and Figure 8, when the other process parameters were the same, the cutting forces in UVAM did not converge to the cutting forces in CM as the feed rate changes. Instead, there was always a certain difference between the cutting forces in UVAM and CM, which indicated that the change in the feed rate had little effect on the ultrasonic vibration effect of UVAM. Using UVAM at different feed rates can still reduce cutting forces during the machining process.

**Figure 9:**
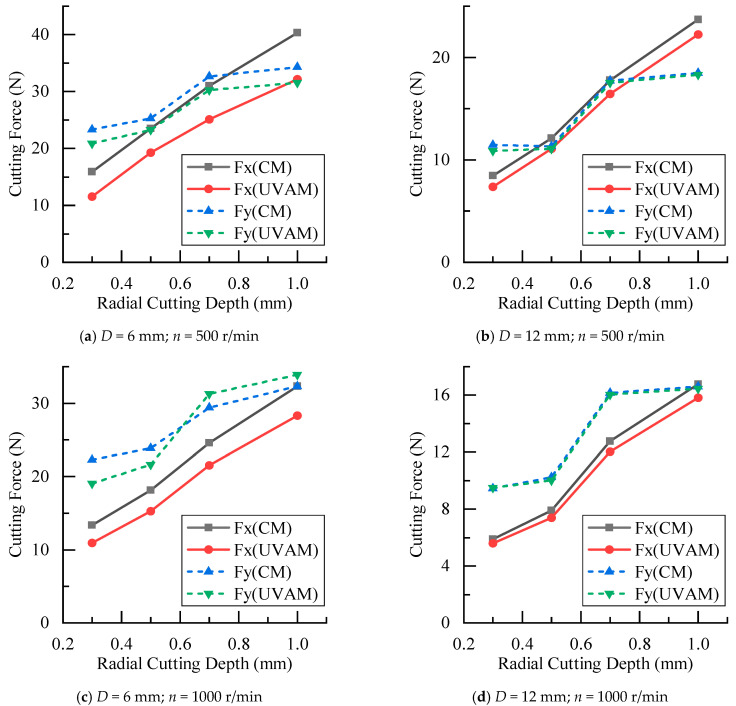
Comparison of cutting forces between CM and UVAM at different radial cutting depths.

**Figure 10:**
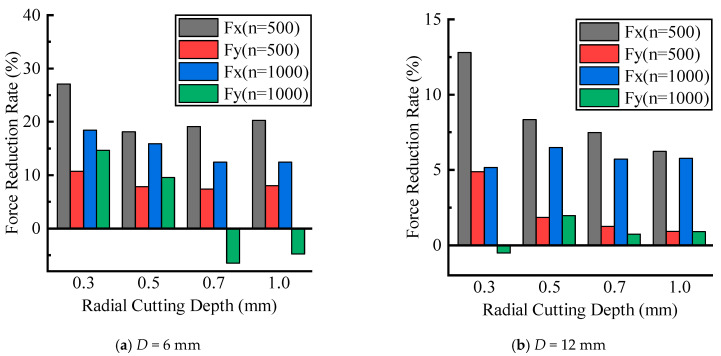
Effect of radial cutting depth on cutting force reduction.

The cutting force experimental results under different radial depths of cut are shown in Figure 9. It can be seen that as the radial cutting depth increased, the cutting forces of CM and UVAM gradually increased. When the process parameters were the same, the cutting force of UVAM was smaller than that of CM. Using Equation (10) to calculate, the cutting force reduction rate under different radial cutting depths was obtained, as shown in Figure 10. In Figure 10, the average cutting force reduction rate for the D=6 mm tool was as follows: at n=500 r/min, $\delta F_{x}=21.1\%,$ and $\delta F_{y}=8.5\%$; at n=1000 r/min, $\delta F_{x}=14.8\%$, and $\delta F_{y}=3.2\%$. For the D=12 mm tool, the average reduction rate was as follows: at n=500 r/min, $\delta F_{x}=8.7\%$, and $\delta F_{y}=2.2\%$; at n=1000 r/min, $\delta F_{x}=5.8\%$, and $\delta F_{y}=0.8\%$. It can be observed that when the tool diameter and spindle speed were the same, which meant the duty cycle was the same, the cutting force reduction rates obtained from the radial cutting depth experiment were similar to those obtained from the feed rate experiment. This indicated that when the duty cycle remained constant, the effect of UVAM in reducing the cutting force did not show significant variation. The experimental results showed the same trend as in Figure 8; when the process parameters were the same, the cutting force reduction rate of the D=6 mm tool was higher than that of the D=12 mm tool, and under the same tool diameter, the cutting force reduction rate at n=500 r/min was higher than that at n=1000 r/min.

According to Figure 9 and Figure 10, it can be concluded that when other parameters were the same, the change in the radial cutting depth did not make the cutting force of UVAM converge to the cutting force of CM. Instead, there was always a certain difference between the cutting forces in UVAM and CM. This indicated that the variation in radial cutting depth had little effect on the cutting force reduction effect of UVAM.

**Figure 11:**
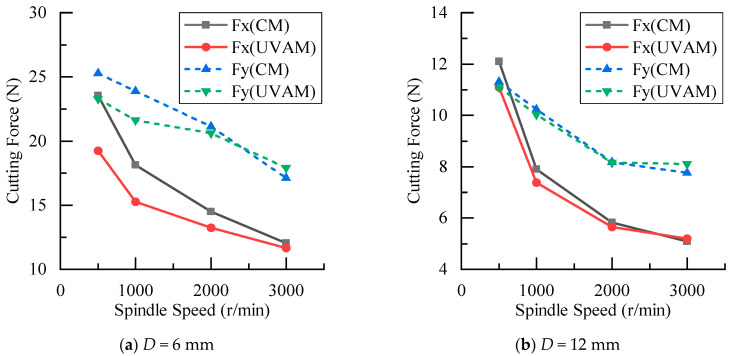
Comparison of cutting forces between CM and UVAM at different spindle speeds.

**Figure 12:**
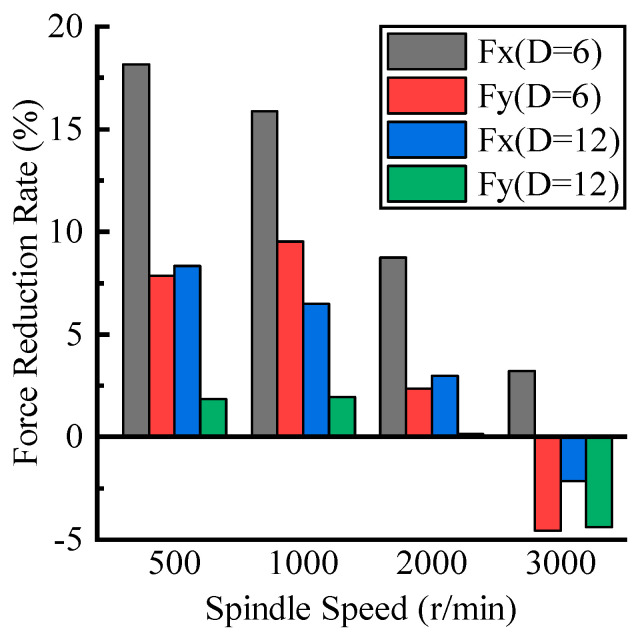
Effect of spindle speed on cutting force reduction.

The experimental results of cutting forces at different spindle speeds are shown in Figure 11. It can be seen that as the spindle speed increased, the cutting force gradually decreased. In most cases, the cutting force in UVAM was smaller than that in CM. However, as the spindle speed increased, the cutting force of UVAM gradually approached the cutting force of CM, until at *n* = 3000 r/min, the cutting force of UVAM was close to or greater than the cutting force of CM. Figure 12 is calculated through Equation (10), it shows the decrease in the cutting force reduction rate as the spindle speed increased. At n=3000 r/min, both tools with different diameters struggled to reduce the cutting force of the machining process by using UVAM. At this time, the duty cycle was 1, and the cutting force reduction rate ranged from 3.2% to −4.6%. Abootorabi et al. [1] obtained similar results, that is, as the spindle speed increased, the cutting force of UVAM approached that of CM. This indicated that the spindle speed affected the reduction in the cutting force by UVAM, and the lower the spindle speed, the lower the duty cycle, and the better the effect of using UVAM to reduce the cutting force. If the spindle speed was too high, UVAM could not effectively reduce the cutting force.

The process parameters used for plotting Figure 5e were the same as those in the experiment of this work. Therefore, as shown in Figure 5e, in this experiment, the tool with a diameter of D=12 mm only had the duty cycle DC<1 at the spindle speed of n=500 r/min, while the tool with a diameter of D=6 mm had the duty cycle of DC=1 only at the spindle speed of n=3000 r/min. This indicated that compared with the D=12 mm tool, when using the D=6 mm tool for the experiment, there were more occurrences of tool–workpiece separation, and under the same process parameters, the duty cycle of the D=6 mm tool was smaller, resulting in a longer tool–workpiece separation time.

**Figure 13:**
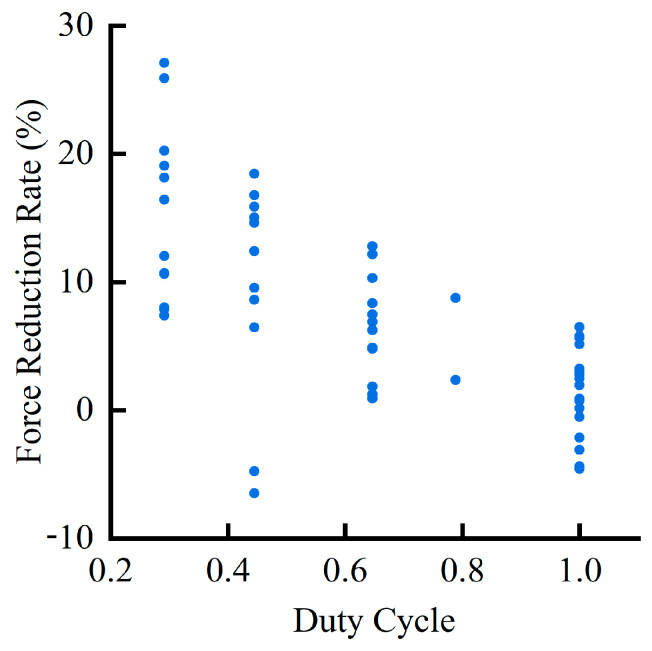
Relationship between duty cycle and cutting force reduction rate.

All the experimental data in this work were plotted to show the relationship between the duty cycle and the magnitude of the cutting force decrease, as shown in Figure 13. It can be observed that as the duty cycle increased, the proportion of the tool–workpiece separation time to the total machining time decreased. The tool–workpiece contact time of UVAM gradually approached that of CM. The cutting forces of UVAM and CM also gradually approached each other, and the cutting force reduction rate in UVAM gradually decreased. Ni et al. [19] reached a similar conclusion in their study, they found that a shorter tool–workpiece contact time can reduce the cutting force. When D=6 mm and n=500 r/min, the duty cycle DC=0.2916; at this time, the cutting force in UVAM was 7.4% to 27% lower than that in CM. When the duty cycle was DC=1, the cutting force in UVAM was −4.5% to 7.5% lower than that in CM.

Based on the analysis above, we can draw the following conclusions: the duty cycle, which represents the proportion of the tool–workpiece contact time to the total machining time, has an influence on the effect of UVAM in reducing the cutting force. The smaller the duty cycle, the longer the tool–workpiece separation time, allowing UVAM to significantly reduce the cutting force. Conversely, a larger duty cycle, indicating a shorter tool–workpiece separation time, results in a diminished effect of UVAM in reducing the cutting force. Therefore, to achieve the effect of reducing the cutting force in UVAM and make it more obvious, it should be considered to have a smaller duty cycle during machining. This can be achieved by using a smaller spindle speed and tool diameter and a larger tool helix angle, ultrasonic amplitude, and ultrasonic frequency while meeting the machining requirements.

## 5. Conclusions

In this work, a method for calculating the duty cycle in UVAM was provided, which was used to analyze the tool–workpiece separation in UVAM. An experimental study on the cutting force of milling CFRP was conducted to compare the cutting forces between UVAM and CM, and the relationship between the duty cycle and the UVAM cutting force was analyzed. The following conclusions were drawn:

1. UVAM significantly reduces the cutting force during CFRP machining. In this study, UVAM achieved a maximum reduction of 27% in the cutting force.
2. The reduction rate of the cutting force in UVAM is related to the duty cycle. A larger duty cycle results in a shorter tool–workpiece separation time, which leads to UVAM and CM having closer cutting forces and a smaller reduction rate in the cutting force.
3. The duty cycle is influenced by the spindle speed, tool diameter, tool helix angle, ultrasonic amplitude, and ultrasonic frequency. Increasing the spindle speed and tool diameter will increase the duty cycle, while increasing the tool helix angle, ultrasonic amplitude, and ultrasonic frequency will decrease the duty cycle.
4. To maximize the effect of UVAM in reducing the cutting force, it is recommended to reduce the duty cycle while ensuring the requirements of the machining process parameters. This can be achieved by reducing the spindle speed or tool diameter and increasing the tool helix angle, ultrasonic amplitude, or ultrasonic frequency.

This study mainly focused on the duty cycle and cutting force of UVAM. In future research, further investigation will be conducted to explore the mechanism of reducing the cutting force in UVAM and establish a cutting force model for UVAM. In addition, for the application of UVAM in CFRP machining, it is also needed to study the impact of UVAM on the surface quality of CFRP and to explore the role of UVAM in reducing the surface roughness and minimizing machining damage.

## Figures and Tables

**Table 1:** Process parameters in the experiment.

Number	$v_{f}\;\left(\mathrm{mm}/\mathrm{mim}\right)$	$a_{e}\;\left(\mathrm{mm}\right)$	$n\;\left(r/\min\right)$
1	50, 100, 200	0.5	500, 1000
2	100	0.3, 0.7, 1	500, 1000
3	100	0.5	2000, 3000

**Table 2:** Cutting force measurement data.

**No.**	** $v_{f}$ ** ** (mm ** ** /min) **	** $a_{e}$ ** ** $\left(mm\right)$ **	** n ** ** (r/min) **	** $F\left(D\;=\;6\;mm\right)\left(N\right)$ **				** $F\left(D\;=\;12\;mm\right)\left(N\right)$ **			
				**Fx**		**Fy**		**Fx**		**Fy**	
				CM	UVAM	CM	UVAM	CM	UVAM	CM	UVAM
1	50	0.5	500	18.64	13.81	25.73	22.99	6.08	5.91	9.46	9.75
2	100	0.5	500	23.52	19.25	25.27	23.28	7.90	7.39	10.23	10.03
3	200	0.5	500	32.67	27.30	30.35	26.70	12.22	11.53	12.51	12.20
4	50	0.5	1000	15.56	12.95	24.85	22.71	5.02	4.83	9.34	9.29
5	100	0.5	1000	18.14	15.26	23.89	21.61	5.83	5.66	8.18	8.16
6	200	0.5	1000	22.74	19.32	29.11	27.23	9.42	9.29	11.35	11.39
7	100	0.3	500	15.87	11.57	23.35	20.85	8.46	7.38	11.45	10.89
9	100	0.7	500	31.03	25.11	32.68	30.26	17.77	16.44	17.74	17.52
10	100	1	500	40.33	32.16	34.32	31.57	23.71	22.23	18.50	18.33
11	100	0.3	1000	13.39	10.92	22.29	19.03	5.90	5.59	9.45	9.50
12	100	0.7	1000	24.57	21.52	29.39	31.29	12.77	12.04	16.15	16.03
13	100	1	1000	32.31	28.30	32.34	33.88	16.78	15.81	16.60	16.45
14	100	0.5	2000	14.51	13.24	21.12	20.62	5.83	5.66	8.18	8.16
15	100	0.5	3000	12.05	11.66	17.12	17.90	5.09	5.20	7.77	8.11

## Data Availability

All data and materials used or analyzed during the current study are included in this manuscript.
